# A Robust Artificial Intelligence Approach with Explainability for Measurement and Verification of Energy Efficient Infrastructure for Net Zero Carbon Emissions

**DOI:** 10.3390/s22239503

**Published:** 2022-12-05

**Authors:** Harsha Moraliyage, Sanoshi Dahanayake, Daswin De Silva, Nishan Mills, Prabod Rathnayaka, Su Nguyen, Damminda Alahakoon, Andrew Jennings

**Affiliations:** Centre for Data Analytics and Cognition, La Trobe University, Melbourne, VIC 3086, Australia

**Keywords:** artificial intelligence, Energy Conservation Measures (ECM), Measurement and Verification (M&V), explainability AI (XAI), energy efficiency, baseline modeling

## Abstract

Rapid urbanization across the world has led to an exponential increase in demand for utilities, electricity, gas and water. The building infrastructure sector is one of the largest global consumers of electricity and thereby one of the largest emitters of greenhouse gas emissions. Reducing building energy consumption directly contributes to achieving energy sustainability, emissions reduction, and addressing the challenges of a warming planet, while also supporting the rapid urbanization of human society. Energy Conservation Measures (ECM) that are digitalized using advanced sensor technologies are a formal approach that is widely adopted to reduce the energy consumption of building infrastructure. Measurement and Verification (M&V) protocols are a repeatable and transparent methodology to evaluate and formally report on energy savings. As savings cannot be directly measured, they are determined by comparing pre-retrofit and post-retrofit usage of an ECM initiative. Given the computational nature of M&V, artificial intelligence (AI) algorithms can be leveraged to improve the accuracy, efficiency, and consistency of M&V protocols. However, AI has been limited to a singular performance metric based on default parameters in recent M&V research. In this paper, we address this gap by proposing a comprehensive AI approach for M&V protocols in energy-efficient infrastructure. The novelty of the framework lies in its use of all relevant data (pre and post-ECM) to build robust and explainable predictive AI models for energy savings estimation. The framework was implemented and evaluated in a multi-campus tertiary education institution setting, comprising 200 buildings of diverse sensor technologies and operational functions. The results of this empirical evaluation confirm the validity and contribution of the proposed framework for robust and explainable M&V for energy-efficient building infrastructure and net zero carbon emissions.

## 1. Introduction

A global transition into net zero carbon emissions has been accepted, and in some cases mandated, by governments, organizations, and concerned communities as the critical and pragmatic solution to climate change. An increase in renewables generation and a decrease in energy consumption delivers climate action, as well as energy sustainability and reduced operational costs. Governmental policies on energy efficiency are aimed at supporting climate action, such as the Energy Policy Act (EPA), an executive order passed by the US government to improve the energy efficiency in 15% of the buildings by 2015 with respect to the 2003 baseline and the US Climate Bill 2022 proposed investment worth nearly $370 billion towards energy efficiency and climate change reduction efforts [1,2]. The Energy Efficiency Directive by the European Parliament has the objective of reducing greenhouse gas emissions by 55% compared to 1990 levels to achieve climate neutrality in 2050 and improve energy efficiency across all industries by 9% compared to 2020 levels [3]. Building infrastructure is a large contributor to carbon emissions, at more than 30% of the energy consumption demand and 25% of global greenhouse gas emissions [4]. Energy Conservation Measures (ECM) are a formal approach to reducing the energy consumption of building infrastructure. ECM leverages advanced sensor technologies and instrumentation to measure, record, and validate consumption in real time. These sensor technologies must be consolidated with computational methods for effective analysis and interpretation that drives insight generation and decision-making capabilities. Some examples of ECM initiatives on building infrastructure are retrofitting heating, ventilation, and air conditioning (HVAC) units, LED lighting replacement, and behavior modification of human operators and users of the building spaces [5].

Measurement and Verification (M&V) protocols are a repeatable and transparent methodology to evaluate such ECM initiatives. M&V can be simplified as the calculation of the energy saved (or not consumed) due to the efficiency of the ECM initiative [5,6,7]. As savings cannot be directly measured, it is determined indirectly as the difference between pre-retrofit and post-retrofit consumption, while controlling for all other variables that may influence consumption [8,9]. International Performance Measurement and Vitrification Protocol (IPMVP) [10] and the American Society of Heating, Refrigerating and Air-Conditioning Engineers (ASHRAE) Guidelines 14 [11] are established methodologies for quantifying the M&V of ECMs [12,13]. These guidelines estimate the savings of ECM using a baseline energy usage model built on pre-ECM data and then compare this with post-ECM usage [14]. IPMVP provides four methods (known as options A, B, C, and D) for M&V analysis and reporting. Options A and B are prescribed for reporting on a specific isolated ECM, while C and D are prescribed for M&V reporting at the building level. The latter is more common as ECM initiatives are focused on building infrastructure and their impact on emissions and costs. However, both options require energy usage data for the entire building for more than one year to build reliable regression models that can estimate savings of more than 10% [10,15,16]. This requirement of one year of data is a significant limitation impacting the adoption of IPMVP, primarily because buildings undergo multiple ECM initiatives within a period of one year, and while in operation, buildings can be shut down for routine maintenance and/or unexpected events, such as working-from-home measures enacted during the COVID-19 pandemic. Further motivation for M&V reporting in short-time intervals is the need for quick cycles of informed decision-making that evaluate the cost-efficiency and performance of M&V projects for future ECM initiatives and the early identification of underperforming projects [17]. To the best of our knowledge, the only related work in this space is Effinger et al. [18], who proposed and demonstrated the use of two separate baseline models for pre-ECM and post-ECM, where the post-ECM model was developed using 3, 6, 9 and 12 months, and then both models were projected over a common base such as TMY temperature data of one year to estimate annual savings. However, this approach leads to a high degree of variability in the ECM calculation. In this paper, we address this limitation by proposing an artificial intelligence (AI) approach that is robust to this variability and incorporates explainability of the predictive outcome for effective M&V of ECM initiatives. This approach is more robust than the method of two separate models proposed by Effinger et al. [18] because we build a single model for both pre- and post-ECM energy consumption that is then applied over a common base, such as TMY temperature data, to estimate annual savings. This ensures the predictive model takes the maximum benefit of shorter time interval data to detect granular pre- and post-ECM energy consumption patterns of buildings in a single model as well as captures the impact of the seasonality. Our proposed approach contributes to the recent algorithmic and analytical advancements of AI in several application domains, including savings estimation, energy forecasting, grid optimization, renewal energy, demand prediction, and system planning [19,20,21,22].

## 2. Materials and Methods

**Figure 1:**
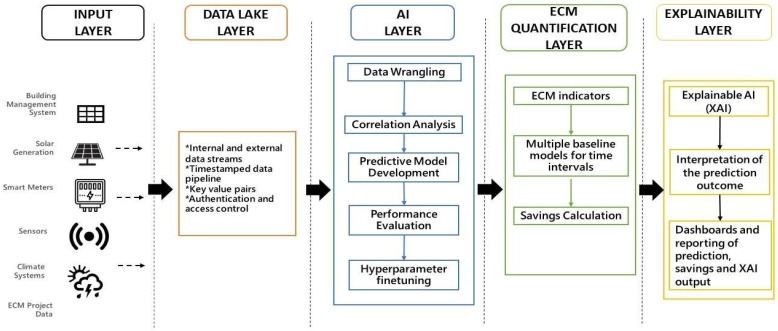
Proposed AI M&V savings estimation framework.

The proposed AI approach is illustrated in Figure 1. It is composed of five vertical layers, they are, the input layer, data Lake layer, artificial intelligence (AI) layer, ECM quantification layer, and finally, the explainability layer. The input layer receives data streams from the building management systems (BMS), solar photovoltaic (PV) systems, smart meters, sensors, climate systems, and ECM project management database. These data streams are extracted, transformed, and loaded into a data lake which forms the second vertical layer. This data lake is accessed by the AI layer which pre-processes and formulates relevant attributes to build the baseline consumption model and finetune its hyperparameters. The AI layer ensures robustness to the variability of the ECM calculations by using a single predictive model to detect pre- and post-ECM energy consumption patterns, which are then used to estimate annual savings. This single model approach can be applied to short time intervals so that granular ECM patterns and the impact of seasonality is determined using low volumes of data. XGBoost, the supervised learning algorithm used to build the predictive model, adds a further level of robustness through its boosting and meta-learning properties. The fourth layer for ECM Quantification evaluates the performance of the model, and then computes and quantifies the ECM savings. Finally, the fifth layer applies Shapley Additive exPlanations (SHAP) game theoretic approaches to explain and interpret the model and its M&V outcomes and communicates the data-driven decision-making insights on M&V of ECM initiatives using interactive analytics dashboards. The following subsections delineate each of the post-input verticals, in terms of their constituents and functionality.

### 2.1. Data Lake Layer

The data lake layer is formally defined as a repository for “all” types of data generated by multiple systems and functions within an organization [23,24,25]. Data lakes are advantageous primarily due to the separation of data produced by systems (processes or entities) from data consumed by humans and systems, as well as their adaptable structure in providing a storage layer for analytics/AI insights and data-driven decision-making [26]. It receives internal and external data from databases, data warehouses, raw data streams, and conventional repositories, such as flat files and spreadsheets. Data formats are not defined until the point of utility, which ensures the data can be stored, managed, and leveraged in an adaptable manner, typically using the key-value pairs format. The data lake is updated regularly with timestamps for when the data was received, in addition to the timestamps for when the data was created and/or modified in the source pipelines and storage. The data lake can also be used directly for data visualization and search functions that are required by ECM audits and management activities.

### 2.2. Artificial Intelligence (AI) Layer

The AI layer retrieves relevant data from the data lake and begins with a data wrangling phase that evaluates data quality. This is an important first phase because energy data streams contain missing and erroneous due to reasons such as sensor failures, network failures, and data transmission issues. Descriptive statistical analysis and visualization techniques, such as histograms and boxplots can be used to identify these issues. For identified issues, we have used the mean value imputation method to remove variables with more than 5% of missing values and impute the values of variables with less than 5% missing values. Following this preprocessing phase, we identify appropriate variables to build the AI models. Correlation analysis between independent variables and dependent variables is used to identify highly correlated variables, while we also assess correlation among independent variables to detect and avoid multicollinearity.

The AI algorithms and models we have leveraged in this approach align with the guidelines specified in IPMVP and ASHRAE. Firstly, we have introduced a parameter to represent ECM activity between the pre-retrofit period and post-retrofit periods. This ensures the AI model learns the change in energy consumption before and after the ECM project. For the learning task, we used the XGBoost algorithm. Boosting is an established machine learning concept with algorithms, such as adaptive boosting (AdaBoost), boosting tree, gradient boosting (GB), Extreme gradient boosting (XGBoost) and Light gradient boosting machine (lightGBM). XGBoost is a formally established supervised learning algorithm for predictive model development due to its accuracy and performance as evidenced by several studies in the energy domain itself [12,15,27,28]. A comparative analysis conducted by Cabrera et al. [29] showed that XGBoost has the highest accuracy in terms of CV(RMSE) compared to linear and symbolic regression models. Boosting focuses on predictions with high error in the initial training step by adjusting the sample distribution for the next training step. This process continues for multiple iterations until the number of learners reaches a stopping criterion. Boosting models build a set of weak learners to obtain strong learner with better performance compared to individual weak learners.

Most machine learning models with high accuracies are subject to over-fitting where the model becomes optimized for the training data, thereby performing poorly in testing data and live applications. K-fold cross-validation is an effective method to detect and measure over-fitting in machine learning models; this method randomly splits the training dataset into k subsets, which are called folds, with the same sizes. Since limited data is used in our approach with shorter pre- and post-ECM periods, we have applied 10-fold cross-validation. A further optimization of the machine learning model that improves performance is to finetune the hyperparameters of the learning algorithm. In XGBoost, the relevant hyperparameters are: (1) learning rate which provides the shrinkage at each time step, (2) estimators that provide the number of weak learners or the regression trees in the model and (3) tree depth for the number of splits during training [30]. The broad range of values that each hyperparameter takes and the number of such parameters that can be finetuned increases the computational complexity of the model development phase. This is typically addressed using the grid search method, which train models per all the hyperparameter combinations to find the most effective configuration or randomized search, which randomly select hyperparameter combination [31].

The robust evaluation of baseline accuracy is critical for M&V use cases as they aim to capture the quality of each ECM initiative [32]. Therefore, in our framework, we utilize the large volumes of smart meter data accumulated in most ECM settings to evaluate the performance of the XGBoost models built in the previous layer. Given the disposition of the performance evaluation task, the following metrics are the most effective; Normalized Mean Bias Error (NMBE), the Coefficient of Variation of the Root Mean Squared Error (CV(RMSE)), Coefficient of Determination (R^2^) and Mean Absolute Percent Error (MAPE). The mathematical formulations of NMBE, CV(RMSE), R^2^, and MAPE are presented in Equations (1)–(4), where ${\hat{y}}_{i}$ is the predicted value, $y_{i}$ is the actual meter reading, $\bar{y}$ is the average of $y_{i}$ and n is the total number of data points.

(1) $$NMBE=\frac{\frac{1}{n}\sum_{i}^{n}\left(y_{i}-{\hat{y}}_{i}\right)}{\bar{y}}\;*100$$

(2) $$CV\left(RMSE\right)=\frac{\sqrt{\frac{1}{n}\sum_{i}^{n}{\left(y_{i}-\hat{y_{i}}\right)}^{2}}}{\bar{y}}\;*100$$

(3) $$R^{2}=1-\frac{\sum_{i}^{n}{\left(y_{i}-\hat{y_{i}}\right)}^{2}}{\sum_{i}^{n}{\left(y_{i}-\bar{y}\right)}^{2}}\;$$

(4) $$MAPE=\frac{1}{n}\sum_{i}^{n}\frac{\left|y_{i}-\hat{y_{i}}\right|}{y_{i}}$$

According to the ASHRAE Guideline 14 uncertainty analysis of the M&V predictive model provides a degree of confidence in the actual value when measurement procedures are applied. CV(RMSE) measure is a prominent metric in ASHRAE Guideline 14 and IPMVP where the requirement is a prediction model with a CV(RMSE) value of less than 30, while IPMVP requires a value less than 20 CV(RMSE) [10,11].

### 2.3. ECM Quantification

The fourth vertical focuses on the savings quantification of the ECM initiative. Most quantification approaches follow the standard method defined in the IPMVP guidelines (Equation (5)), which includes an adjustment factor along with the baseline and post-retrofit energy usage. We propose a robust quantification method that is facilitated by the design of the predictive model in the AI layer. The binary ECM indicators introduced into the predictive model are used to estimate the impact of the ECM project by revising the values of the introduced features. For example, if the predictive model is built using consumption data and weather data from 2019, then the introduced binary ECM indicator ‘is_ecm’ is used to reflect the impact of ECM installation. During the model training time, this feature value will be set based on the ECM installation date to feed the pre and post-retrofit energy consumption behavior of the building to the model. Then, to evaluate the yearly savings with estimated weather data from a future or current year (noting that annual weather data typically lies within a narrow range across adjacent years), we set the introduced binary feature ‘is_ecm’ value to True and estimate energy consumption for the year assuming ECM event in place for the entire year. Next, we set the value to False, to which the model provides the estimated annual consumption without any ECM project for the entire year.

As noted in Equation (6), the difference between the sum of the energy consumption without the event and the sum of the energy consumption with the event over the year provides the annual energy savings for that ECM project. The savings percentage can be calculated using Equation (7) to estimate the overall energy savings percentage of the ECM project. The details of the savings quantification process are shown in Algorithm 1.

(5)  Savings=Baseline Energy Use−PostRetrofit Energy Use ± Adjustments

(6)  Savings=Sum of Consumption Without Event−Sum of  Consumption With Event

(7) $$Savings\;Percentage=\frac{Consumption\;Without\;Event-Consumption\;With\;Event}{Consumption\;Without\;Event}*100\;$$

**Algorithm 1:** Savings Quantification
**Input:**
*X*: Training Data *features*: Selected set of features *Y*: Training Energy Consumption *E*: ECM Projects *R*: Reporting Period Data
**Output:**
*Z***:** Savings dictionary1:
*model ← XGBoost()*
2:*H ←* initialize hyperparameters dictionary3:
*Z = {}*
4:
**for**
*each ECM*
*E_i_ ∈ E*
**do**
5:

 *X[E_i_ [‘name’]] ← Add new feature per ECM project and set default value to 0*
 *R[E_i_[‘name’]] ← Add new feature per ECM project*
6:
 **for** all data points X_j_
*∈ X **do***7:

  ***If*** X_j_*[‘date’] > E_i_[‘date_start’] **then***8:

    X_j_*[E_i_ [‘name]] = 1*




  **end if**

9:

 **end for**

10:

 *features.add (E_i_[‘name’])*
11:
**end for**
12:
*optimized_model = GridSearchCV (model, params = H, scoring = ‘rmse’)*
13:
*optimized_model.fit(X[features], Y)*
14:**for** 
*each ECM E_i_*
*∈ E*
**do**
15:
 *R[E_i_[‘name’]] ←* Set feature value to *0 for all data points*16:

 *consumption_no_ecm = optimized_model.predict(R[features])*
17:
 *R[E_i_[‘name’]] ←* Set feature value to *1 for all data points*18:

 *consumption_with_ecm = optimized_model.predict(R[features])*
19:

 *savings = SUM (consumption_no_ecm-consumption_with_ecm)*
20:

 *Z[E_i_[‘name’]] = savings*
21:
**end for**
22:**return** *Z*

### 2.4. Explainability Layer

The explainability layer consists of two key functions, (1) using explainable AI (XAI) methods to interpret the predictive models for M&V and (2) interactive dashboards and reporting of the prediction and XAI outcomes. For the XAI function, the binary ECM indicator features drive the interpretation of the most contributing features in the model. The most widely used XAI method is the SHapley Additive exPlanations (SHAP) framework which is based on the concept of game theory and determines the contribution (or importance) of features and groups of features towards the output of the predictive model [33]. In addition, complex machine learning models such as neural networks and gradient boosting are yet to be adopted widely for M&V estimations despite their high accuracy because of the lack of interpretability [34]. The framework component that explores the model interpretability is a major need for uncovering the model internal behaviors with respect to input features [35]. The second function of the interactive dashboard and reporting aims to unpack the predictive model and its XAI output for further analysis and synthesis. This becomes important in the context of multiple buildings or large organizations where the buildings and the ECM projects need to be evaluated individually and in comparison to other similar projects.

## 3. Empirical Evaluation

We conducted several experiments using the UNICON [36] dataset, which is drawn from the La Trobe Energy AI/Analytics Platform (LEAP), the flagship AI initiative of La Trobe University’s commitment towards achieving Net Zero Carbon Emissions in all campuses by 2029 [37]. There are over 100 buildings from multiple campuses that are classified as academic, accommodation, or administrative. The consumption data is from smart meters and contains 15-min interval energy usage from 2018, 2019, and 2020, where 2020 includes the COVID-19 shutdown period. Weather data is also recorded in 5-min granularity, which includes apparent temperature, dew point temperature, and relative humidity. University buildings reflect similar energy consumption patterns as commercial buildings where energy consumption is higher during afternoons and lower during nights and weekends. The full set of features used in the model is shown in Table 1. The dates of the ECM projects carried out at each of the buildings are shown in Table 2.

### 3.1. Evaluation of AI Model Performance

**Figure 2:**
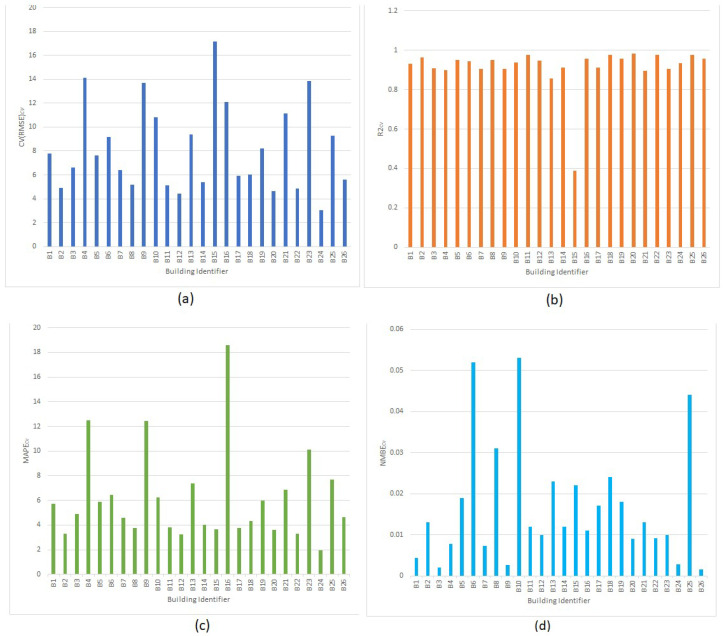
Cross validated XGBoost accuracy results for the year 2018 (**a**) CV(RMSE), (**b**) R^2^, (**c**) MAPE and (**d**) NMBE.

In this section, we evaluate the performance of the models on the selected features from Table 1. For evaluation, we used k-fold cross validation where the dataset is split into k equal segments and for each k-th segment, the model is trained on all other segments, followed by testing on the k-th segment. This repeats k-times and the k results are averaged to produce a single performance metric. This approach is more effective than a singular train-test split because of the multiple runs through which the model is exposed to all the data records instead a single, fixed subset. The results in Figure 2 show the results of 10-fold cross-validation CV(RMSE), R^2^, MAPE, and NMBE mean scores of the models for the buildings. There were no ECM projects for these buildings in the year 2018. We have used yearly 15-min energy consumption data in 2018 to validate the models with cross-validation. As illustrated in Figure 2, cross-validated CV(RMSE) values are within the recommended range specified in IPVMP and ASHRAE, which are 20 and 30, respectively. The R^2^ values in Figure 2 are closer to 1 with majority of the MAPE values are below 10%, while NMBE values are below 0.06 for all the buildings confirms that the variables and dataset used in this experiment are effective at demonstrating the proposed robust and explainable AI approach for ECM calculation.

**Figure 3:**
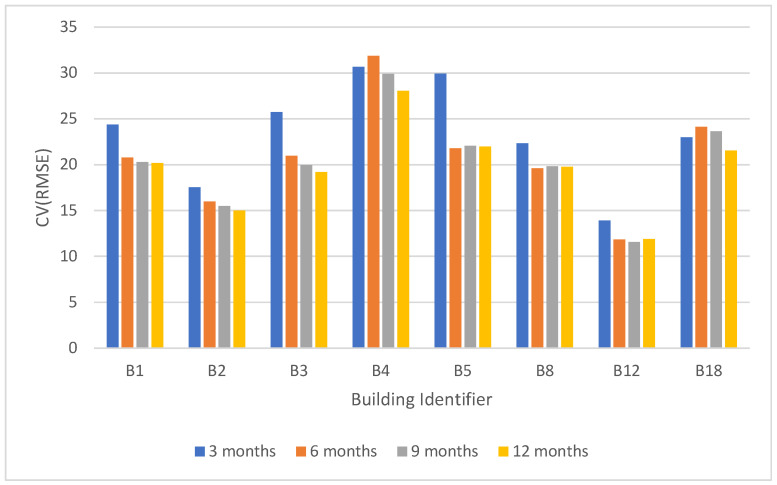
CV(RMSE) values over varying training periods with 1-year prediction period.

The predictive AI models are built using 2018 data and the performance is evaluated using the complete 12 months of 2019 data. The objective of this experiment is to evaluate the impact of using smaller training time durations as existing research studies found that the use of shorter energy interval data provides similar performance measures over longer training periods [15,38]. This will verify the impact of using smaller training durations to build accurate baseline models with our selected set of features. Figure 3 shows the CV(RMSE) values of the models that are trained for 3 months, 6 months, 9 months, and 12 months and evaluated the performance against 12-month training data in 2019. We have only selected buildings that do not have any ECM project during 2019. As noted in Figure 3, models trained on 3 months data performed equally well to the models trained on 6 months, 9 months and 12 months training time periods. This demonstrates the robustness of the proposed approach, where using 3 months’ training data is sufficient for the model to learn energy consumption behavior of the building.

In this dataset, buildings can be categorized into three groups, (1) buildings where the ECM project is a BMS upgrade only, (2) buildings where the ECM project is an LED lighting upgrade only, and (3) buildings with both ECMs. In our proposed method, we have added a binary feature to represent the time periods of before and after the installation date of ECM which is then projected across 2019 to evaluate the energy savings as shown in Figure 3 where the percentage savings is calculated using Equation (7).

### 3.2. Buildings with BMS Upgrade ECM

**Figure 4:**
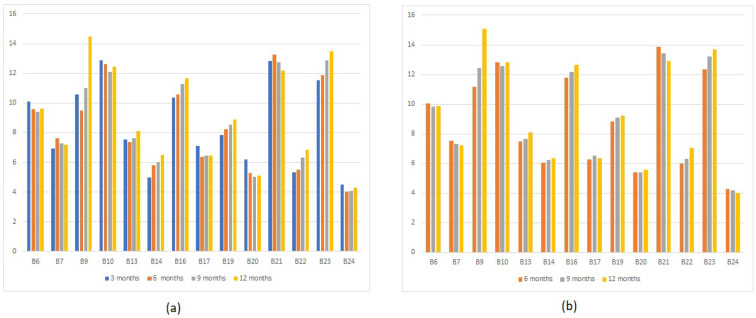
The cross-validated CV(RMSE) values of buildings containing BMS upgrade ECM (**a**) CV(RMSE) values of models generated using 3 months post-retrofit training data (**b**) CV(RMSE) values of models generated using 6 months post-retrofit training data.

We first evaluated the buildings that underwent the BMS upgrade. Model performance is shown in Figure 4 where all CV(RMSE) values are below 15 for all of the buildings, which is within the IPMVP guidelines. Table 3 shows the percentage of savings for the same BMS upgrade measured during different time spans, ranging from 12-, 9-, 6-, and 3-months data prior to the event with 3- and 6-month data after the ECM installation.

**Figure 5:**
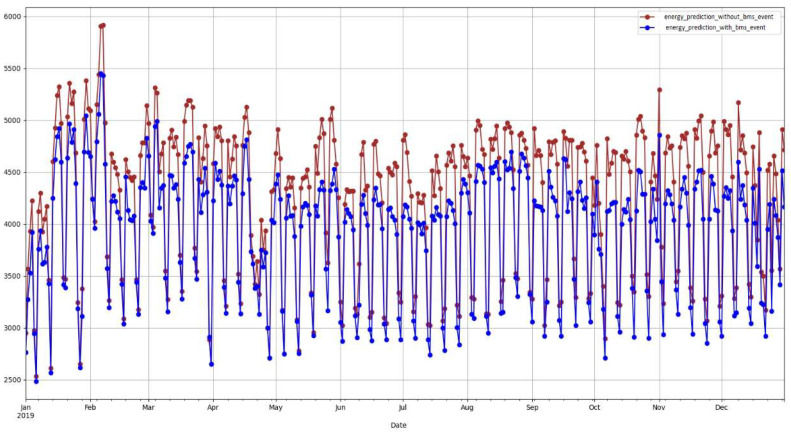
Predicted baselines with and without BMS upgrade event of building B20.

We conducted further on-site analysis of the results presented in Table 3. It was noteworthy that some buildings have lower percentage savings after switching from a 3-month post-event period to a 6-month post-event period. Our on-site analysis revealed that the impact of the BMS upgrade on energy savings reduced over time which is the reason for the reduction of the savings percentage. The percentage savings in the 12 months (post) column is the standard one-year approach of estimating ECM savings where we built a baseline model using a 12-month pre-ECM period and estimate the energy savings percentage compared to actual and adjusted baseline consumption. According to the results, energy savings estimated by the standard approach are higher compared to the proposed method. This is because of the saving percentage estimated by the standard approach is not yearly basis as it is not possible to capture data for 1 year period after the ECM installation due to overlapping ECM projects. The savings calculated using the standard approach also indicates a drop going from 3 months savings to 6 months savings. Hence, the annual savings estimated by our approach is less than the standard method estimation. This indicates the robustness of our approach where it captures the energy savings pattern over time and projects it across the entire year. Using the standard approach, it is not possible to get an estimation of this latent behavior. Figure 5 shows the projection of energy consumption over a year with and without the ECM project. It shows that our approach captures the energy consumption behavior according to the introduced binary feature to reflect the ECM project.

**Figure 6:**
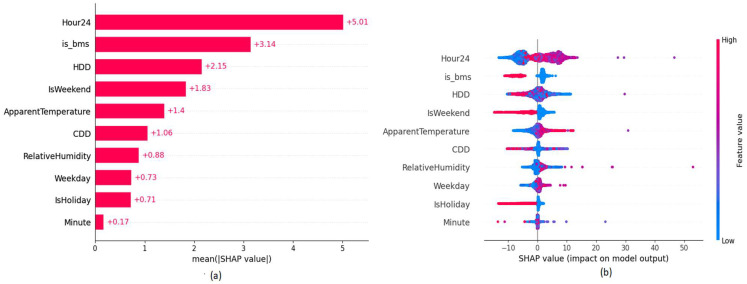
SHAP explainability plots of building B24 (**a**) Variable Importance (**b**) Variable Impact.

In the explainability layer, we studied the models used in the experiments that are based on a 9-month pre-ECM installation period followed by a 3-month post-training period. The variable importance plot in Figure 6 shows the impact of the features based on SHAP values respective to feature values towards the target prediction with descending feature importance. According to the variable importance plot, the ‘is_bms’ binary variable which is related to the BMS upgrade ECM has the second most impact on the target predictions of the model for building B26. SHAP values in the variable impact plot in Figure 6 provide the distribution of the impact of each feature has on the model output. The color represents the feature value where their value goes from low to high from blue to red. As expected, the binary variable ‘is_bms’ has an inverse effect. When the binary value is set to 1, it reduces the energy consumption, while a variable, such as temperature, has a positive impact towards the energy consumption. This indicates that the model has properly captured the impact of the ECM project based on the feature value.

**Figure 7:**
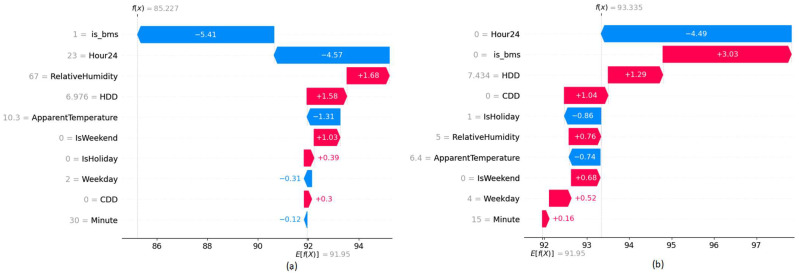
SHAP Waterfall plots of building B24 (**a**) Energy consumption prediction assuming BMS upgrade ECM present (**b**) Energy consumption prediction assuming BMS upgrade ECM absent.

Waterfall graphs shown in Figure 7 are used to analyze the value contribution towards target prediction based on each feature of a given data point. Red color bars indicate an increase while blue color bars indicate a decrease in energy consumption and the length of the bar is proportional to the impact of the feature. When no-ECM is in place that indicates the value zero of the binary variable, the energy consumption increases while it decreases when the value is set to 1. According to the plots, they further verify the model behavior in the present and the absence of the ECM project.

### 3.3. Buildings with LED Retrofit ECM

**Figure 8:**
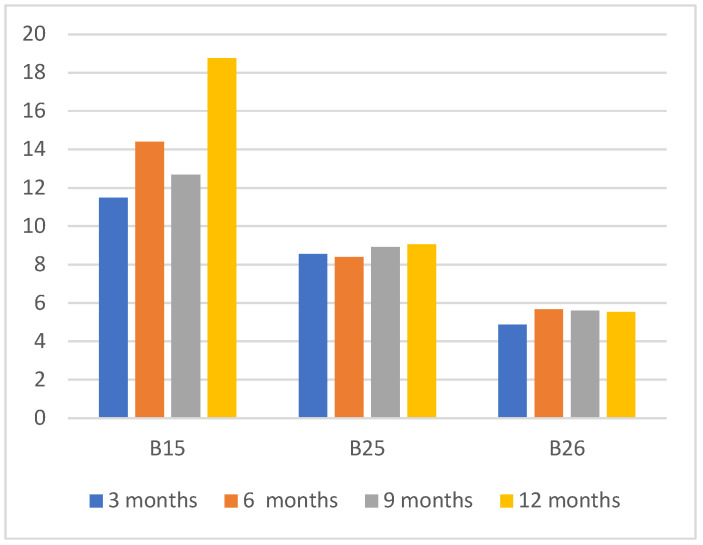
The cross-validated CV(RMSE) values of buildings containing LED retrofit ECM only.

LED retrofit ECM shows consistent energy savings compared to the BMS upgrade. The consistent cross-validated CV(RMSE) results across different pre-retrofit periods shown in Figure 8 are within the acceptable levels of IPMVP guidelines and further validate the consistent impact of LED retrofit ECM. The energy consumption patterns of buildings varied over time and the impact of the BMS upgrade was not consistent across time. This behavior is already observed in our dataset. However, the LED retrofit ECM project has a consistent impact on energy savings. Table 4 shows the percentage of savings for the LED retrofit ECM project that contains only 3 months of data.

**Figure 9:**
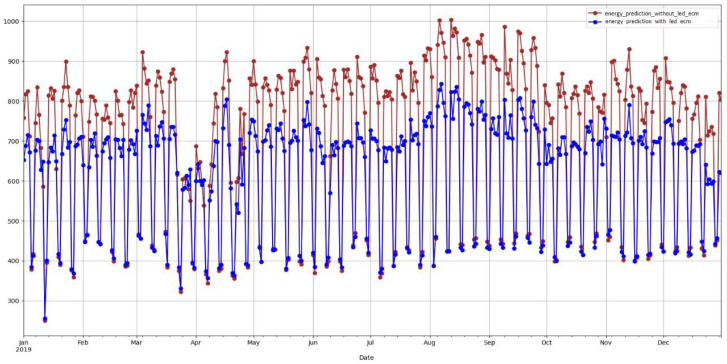
Predicted baselines with and without BMS upgrade event of building B25.

LED ECM saving percentage results in Table 4 show consistent behavior across different selections of pre-retrofit data periods. This indicates that the proposed AI approach identifies consistent energy-saving patterns from the LED retrofit project. Figure 9 shows a consistent gap between the energy consumption of building B16 in the presence and the absence of the LED retrofit project.

**Figure 10:**
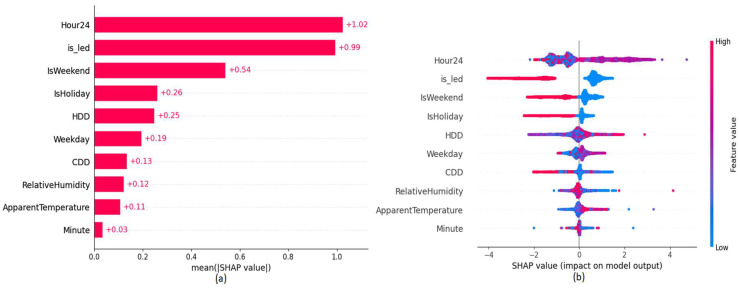
SHAP explainability plots of B26 (**a**) Variable Importance (**b**) Variable Impact.

**Figure 11:**
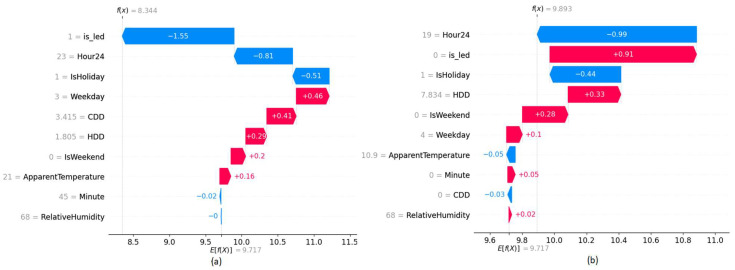
SHAP Waterfall plots of building B26 (**a**) Energy consumption prediction assuming LED retrofit ECM present (**b**) Energy consumption prediction assuming LED retrofit ECM absent.

The explainability plots in Figure 10 and Figure 11, indicate a similar behavior as in BMS upgrade ECM on the introduced ‘is_led’ binary feature to represent the LED retrofit.

### 3.4. Buildings with BMS Upgrade and LED Retrofit ECM

**Figure 12:**
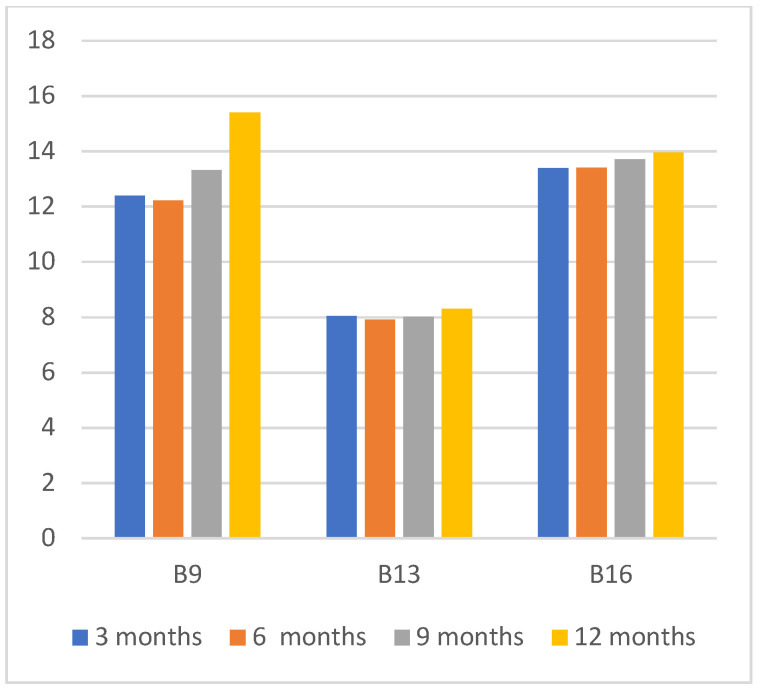
The cross-validated CV(RMSE) values of buildings containing LED retrofit and BMS upgrade ECM.

In this dataset, there are several buildings that were subjected to both BMS upgrade and LED retrofit ECM projects. The performance results of the models trained using the post-retrofit data till the COVID-19 shutdown are shown in Figure 12, which is also within the acceptable level under the standard guidelines. Results of Table 5 were obtained from the buildings that are subjected to both ECM projects. We have evaluated the savings percentage using 3, 6, 9, and 12 months of data prior to the BMS upgrade project.

**Figure 13:**
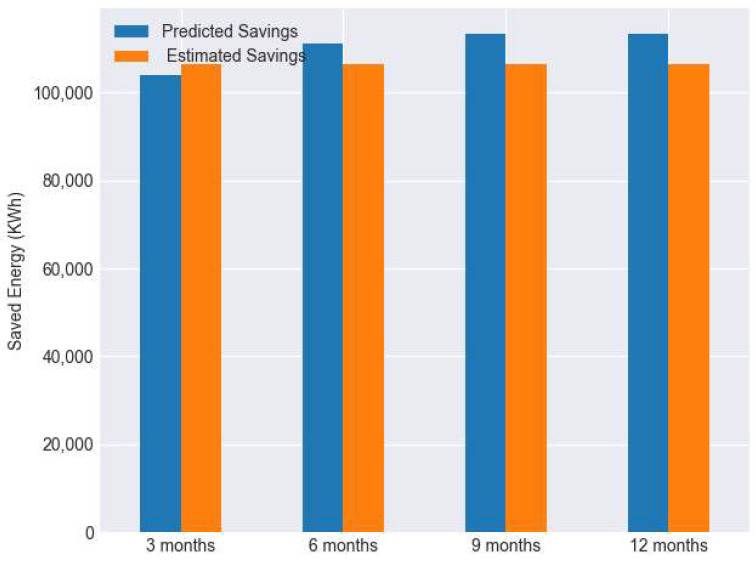
Estimated annual savings vs. calculated annual savings (B15, B16 combined).

We further validated our results with those from the ECM solution provider based on 3000 operational hours, 57.69 per week for buildings B15 and B16. According to the results shown in Figure 13, the estimated savings from our method is similar to the total estimated savings data provided by the vendor. Our method calculates the savings with building operation characteristics, which is more aligned with the proper M&V savings calculation approach.

**Figure 14:**
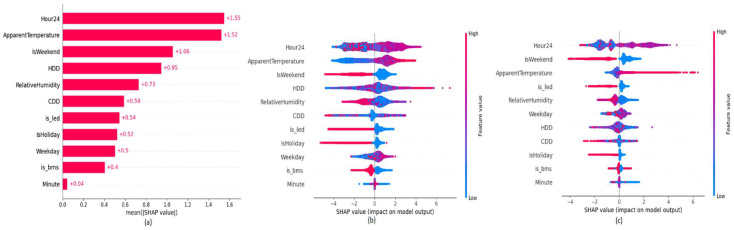
SHAP explainability plots of buildings B9 and B16 (**a**) Variable Importance plot of building B9 (**b**) Variable Impact plot of building B9 (**c**) Variable Impact plot of building B16.

**Figure 15:**
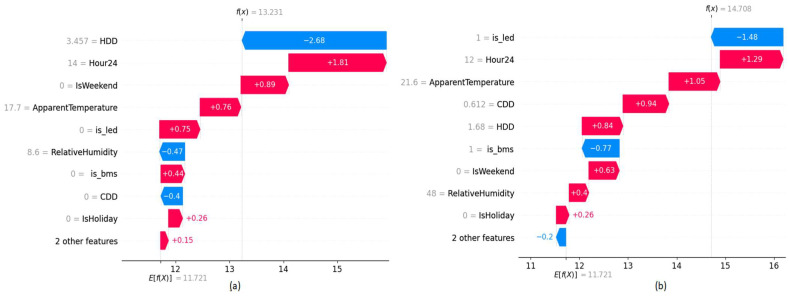
SHAP Waterfall plots of building B9 (**a**) Energy consumption prediction assuming LED retrofit ECM present (**b**) Energy consumption prediction assuming LED retrofit ECM absent.

When a building contains two ECM installations, the introduced two binary variables (is_led, is_bms) show two levels of impact. For building B9, the binary variable, which reflects the LED-retrofit has more impact compared to the binary variable, which reflects the BMS upgrade. We have performed a manual evaluation of the energy consumption behavior after the BMS upgrade and LED retrofit. Our analysis confirmed that the impact of LED retrofit in building B9 has a greater impact compared to the BMS upgrade. Interestingly, when two ECMs projects present in building B9 have a clear inverse impact on both binary variables, which can be observed by the symmetrical separation of colors in the (b) plot of Figure 14. However, the impact of the ‘is_bms’ variable on building B16, which is shown in (c) plot of Figure 14 indicates both positive and negative correlations towards target predictions, while the ‘is_led’ binary feature has a complete inverse relationship. This could indicate that the impact of BMS upgrade degrades over time, while LED retrofit has a complete inverse correlation for energy consumption, which is a permanent event. Figure 15 shows the impact of the introduced features on the model prediction for selected data points.

### 3.5. Impact of ECM Installation Date

**Figure 16:**
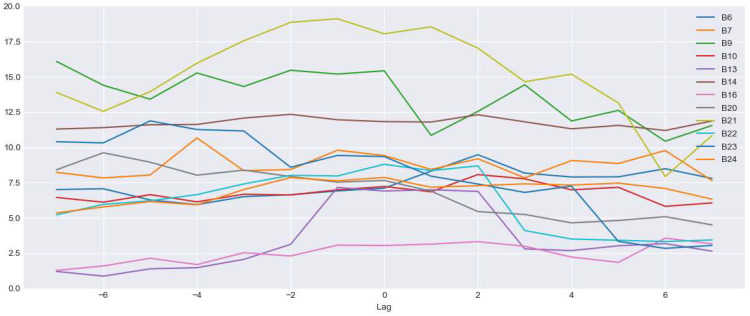
Percentage of savings based on selection of ECM installation date.

Here, we evaluate the selection of the ECM installation date and its impact on the annual savings. Figure 16 shows the impact of percentage savings by selecting 7 days before and after the ECM completion date for the BMS upgrade ECM project. According to Figure 16, several buildings follow a similar decreasing function, but the savings percentage remains mostly consistent across many buildings. The reason for this behavior is that the predictive model observes the reduction of energy savings differently, which changes the impact of the binary feature towards the prediction. Peak percentage savings were observed mostly near the ECM project completion date, which accurately represents the behavior of the building before and after the ECM project.

## 4. Conclusions

In this paper, we have proposed a robust and explainable AI approach for M&V protocols of energy-efficient infrastructure, leading up to a net zero carbon emissions strategy. The proposed approach is novel in its use of small volumes of energy consumption data streams to build effective predictive models for energy savings estimation, as well as its interpretation of the predictive outcome in terms of the variables of the dataset using XAI techniques. The approach consists of five layers: input layer, data lake layer, AI layer, ECM quantification layer, and the explainability layer. We have empirically evaluated the proposed approach on a large real-world dataset of energy consumption data and ECM projects within the multi-campus multi-building setting of La Trobe University. The results representing robustness and explainability of the predictive output and savings estimation confirm the validity and practical value of this AI approach for M&V of ECM projects. As future work, the following limitations of the framework should be addressed; the short time interval for ECM monitoring could contain biased consumption data relating to an event of significance or an outlier, this needs to be factored in as the time interval is incrementally expanded, and the prediction horizon and prediction uncertainty should be incorporated into the model development as further parameters to be finetuned. Experimentation with sophisticated AI models for prediction and further evaluation of the proposed framework across diverse ECM projects in different organizational settings are also recommended. In conclusion, drawing on the proposed framework and its demonstration, it is now timely for policymakers and regulators of energy markets and energy operations to consider the formal adoption of the robust and explainable capabilities of AI for measurement and verification of energy efficient infrastructure for net zero carbon emissions.

## Figures and Tables

**Table 1:** Input variables on ECM projects reported in the UNICON dataset.

Feature	Description
ApparentTemperature	Apparent temperature measured by the weather station
HDD	Heating Degree Days
CDD	Cooling Degree Days
RelativeHumidity	Relative humidity measured by the weather station
Weekday	Binary variable to indicate whether it is a weekday
Hour24	Hour of the day
Minute	Minute of the day
IsHoliday	Binary variable to indicate whether the day is a holiday or not
IsWeekend	Binary variable to indicate whether it is a weekend or not

**Table 2:** ECM projects and installation dates of the university buildings.

	Building Ids	Date
No-CEM	B1, B2, B3, B4, B5	
BMS Upgrade	B6	23 April 2019
	B7	10 May 2019
	B8	15 May 2019
	B9, B10, B11, B12, B13, B14	17 May 2019
	B15, B16, B17, B18, B19	23 May 2019
	B20	29 May 2019
	B21	30 May 2019
	B22	2 June 2019
	B23	7 June 2019
	B24	15 June 2019
LED Installation	B13	21 October 2019
	B25	30 October 2019
	B15	25 November 2019
	B16	23 December 2019
	B9, B26	9 December 2019

**Table 3:** Estimated savings percentage for buildings with BMS upgrade only.

Building	3 Months (Post)				6 Months (Post)				12 Months (Post)	
	3 mths	6 mths	9 mths	12 mths	3 mths	6 mths	9 mths	12 mths	3 mths	6 mths
B9	6.66	11.43	15.43	13.5	3.33	5.62	6.18	5.75	26.41	22.39
B6	7.23	6.07	5.94	6.36	8.52	8.98	6.88	7.11	18.67	21.46
B7	8.05	9.26	9.42	6.89	4.48	4.09	3.26	3.44	−0.69	−4.29
B10	7.38	6.38	6.67	5.6	15.95	13.22	12.44	12.67	8.75	15.59
B13	5.47	7.56	6.9	5.98	3.73	0.72	0.48	1.87	13.85	9.92
B14	11.75	12.89	11.82	11.62	11.58	13.49	12.3	11.6	13.78	15.57
B16	9.25	4.12	2.28	3.49	8.38	2.71	0.72	0.43	0.65	3.75
B17	1.87	0.8	0.36	0.82	0.75	−0.22	−0.26	−0.29	2.23	1.75
B20	5.85	6.14	7.65	8.46	1.48	1.43	2.23	2.04	9.56	4.22
B21	15.67	19.01	18.05	23.04	18.06	16.81	16.03	16.23	29.84	27.28
B22	9.71	8.59	8.8	7.79	2.45	1.34	1.85	1.41	8.92	3.41
B23	12.57	11.2	9.34	7.72	9.87	7.05	6.24	6.66	15.85	12.33
B24	7.07	6.71	7.87	6.82	4.99	4.98	5.4	4.66	11.7	9.06
**Mean**	**8.35**	**8.47**	**8.50**	**8.31**	**7.20**	**6.17**	**5.67**	**5.66**	**12.27**	**10.96**

**Table 4:** Estimated percentage savings for the LED retrofit project.

	3 Months (Post)				Standard
Model	3 Mths	6 Mths	9 Mths	12 Mths	3 Mths
B26	24.93103	26.19834	27.32977	27.96718	26.53
B15	9.4123	9.99353	10.05227	9.29150	10.49
B25	13.63631	13.9096	12.08431	10.82881	11.53

**Table 5:** Estimated percentage savings for LED and BMS upgrade projects.

Building	BMS Upgrade				LED Installation			
	−3 m/3 m	−6 m/3 m	−9 m/3 m	−12 m/3 m	−3 m/3 m	−6 m/3 m	−9 m/3 m	−12 m/3 m
B9	4.77	6.60	8.04	7.78	18.06	15.61	15.54	14.97
B16	10.26	6.42	3.72	3.81	25.11	26.81	27.68	29.96
B13	1.27	1.28	0.60	0.35	0.87	1.43	2.20	2.22

## Data Availability

This study did not report any data; it is uses data already published as the UNICON dataset.
